# EHMTI-0389. Occipital nerve stimulation in the treatment of chronic migraine: experiences of two years

**DOI:** 10.1186/1129-2377-15-S1-M5

**Published:** 2014-09-18

**Authors:** A Göbel, C Göbel, A Heinze, K Heinze-Kuhn, I Petersen, C Meinecke, S Clasen, D Rasche, HM Mehdorn, H Göbel

**Affiliations:** 1 Department of Neurology, University Hospital Schleswig Holstein Campus Lübeck, Lübeck, Germany; 2 Department of Neurology, University Hospital Cologne Germany, Köln, Germany; 3 Kiel Migraine and Headache Centre, Kiel Pain Centre, Kiel, Germany; 4 Department of Neurosurgery, University Hospital Schleswig Holstein Campus Lübeck, Lübeck, Germany; 5 Department of Neurosurgery, University Hospitals Schleswig-Holstein Campus Kiel, Kiel, Germany

## Background

Occipital nerve stimulation (ONS) as a special form of neuromodulation has proven to be an important method in the treatment of therapy refractory chronic migraine.

## Objective

Severely affected patients, not responding to other treatment options, present in our specialised headache centre and treatment network to put up the medical indication for ONS. The patient characteristics and satisfaction rates are analysed over the time span of two years.

## Methods

Descriptive analysis of patient characteristics, therapy satisfaction rates and disability due to migraine of patients treated with ONS since November 2011.

## Results

ONS was used in a total of 43 patients due to therapy-resistant migraine (age 44.98±10.6 years, range 22-69 years, 36 women, 7 men). Migraine had before existed an average of 28.47±11.5 years. When starting treatment, the average MIDAS score was 129.14±58.73, after one year it dropped to 87.44±45.69 (p<0.001). In the follow-up studies during the time of analysis, patients were asked whether they would choose to be treated with ONS again (answers: yes/ambivalent/no). In the follow up studies 1-6, the following answers were found: (1) 56.1/41.5/2.4; (2) 56.8/32.4/10.8; (3) 51.9/44.4/3.7; (4) 55.6/44.4/0; (5) 58.3/41.7/0; (6) 60.0/20.0/0.

## Conclusion

ONS led to a positive evaluation in more than half of patients treated, despite therapy-resistance beforehand. The burden of chronic migraine can be significantly lifted through ONS. Only a very small fraction of patients would not choose this therapy again.

